# Architecture of thermal adaptation in an *Exiguobacterium sibiricum *strain isolated from 3 million year old permafrost: A genome and transcriptome approach

**DOI:** 10.1186/1471-2164-9-547

**Published:** 2008-11-18

**Authors:** Debora F Rodrigues, Natalia Ivanova, Zhili He, Marianne Huebner, Jizhong Zhou, James M Tiedje

**Affiliations:** 1 Michigan State University, NASA Astrobiology Institute and Center for Microbial Ecology, East Lansing, MI 48824, USA; 2 DOE Joint Genome Institute, Walnut Creek, CA 94598-1604, USA; 3 Institute for Environmental Genomics, Department of Botany and Microbiology, University of Oklahoma, Norman, OK, USA; 4 Michigan State University, Department of Statistics and Probability, East Lansing, MI, USA

## Abstract

**Background:**

Many microorganisms have a wide temperature growth range and versatility to tolerate large thermal fluctuations in diverse environments, however not many have been fully explored over their entire growth temperature range through a holistic view of its physiology, genome, and transcriptome. We used *Exiguobacterium sibiricum *strain 255-15, a psychrotrophic bacterium from 3 million year old Siberian permafrost that grows from -5°C to 39°C to study its thermal adaptation.

**Results:**

The *E. sibiricum *genome has one chromosome and two small plasmids with a total of 3,015 protein-encoding genes (CDS), and a GC content of 47.7%. The genome and transcriptome analysis along with the organism's known physiology was used to better understand its thermal adaptation. A total of 27%, 3.2%, and 5.2% of *E. sibiricum *CDS spotted on the DNA microarray detected differentially expressed genes in cells grown at -2.5°C, 10°C, and 39°C, respectively, when compared to cells grown at 28°C. The hypothetical and unknown genes represented 10.6%, 0.89%, and 2.3% of the CDS differentially expressed when grown at -2.5°C, 10°C, and 39°C versus 28°C, respectively.

**Conclusion:**

The results show that *E. sibiricum *is constitutively adapted to cold temperatures stressful to mesophiles since little differential gene expression was observed between 4°C and 28°C, but at the extremities of its Arrhenius growth profile, namely -2.5°C and 39°C, several physiological and metabolic adaptations associated with stress responses were observed.

## Background

About 80% of Earth's surface is 15°C or colder [1]. Psychrophilic together with psychrotolerant bacteria comprise the cold-adapted microorganisms. These microbes have been isolated and characterized from various environments such as polar sediments and soils, as well as open oceans [2,3]. Psychrotolerant microorganisms are of special interest since they grow at a wide range of temperatures, e.g. between -5 and +40°C [3,4], and tolerate large thermal fluctuations in diverse environments [2,5].

Although cold conditions are prevalent on Earth, a reference set of genomes of Gram-positive Bacteria from cold environments has not been available. Recently, whole genome sequences have been determined for a few cold adapted species: *Listeria monocytogenes *[6], *Colwellia psychrerythraea *34H [7], *Idiomarina loihiensis *L2TR [8], *Pseudoalteromonas haloplanktis *TAC125 [9], and *Psychromonas ingrahamii *37 [10]. Most of the work on these microorganisms has not been devoted in exploring the mechanisms of thermal adaptation over their entire growth temperature range. During thermal adaptation cells undergo many cellular modifications in order to survive and grow at extreme temperatures. A network of genes that are activated simultaneously or in cascade fashion generates these modifications. These genes have not been completely elucidated in psychrotrophs and psychrophilic microoganisms. Most of the studies examining the cold and heat stress responses were done in mesophilic bacteria and a range of mechanisms has been identified. These mechanisms involve preserving the flexibility, topology, and interactions of macromolecules such as DNA, RNA and proteins, maintaining the homeoviscous adaption of the cell membrane, protecting the cell from disruption by producing osmoprotectants, and maintaining the diffusion rate and enzyme kinetics inside the cell [11-13].

The variation in specific growth rate as a function of temperature is commonly portrayed by Arrhenius equations [14]. The Arrhenius profiles of most bacteria are characterized by a linear portion in a 20°C suboptimal growth range, i.e. the normal Arrhenius range [14]. Below and above the normal range, deviations of the thermo-dependence of growth from the Arrhenius law reveals the inability of cells to maximize their growth rate [15], but little is known about the reasons why microbes are unable to maximize their growth rate at divergent temperatures.

In the psychrotrophic bacterium *Exiguobacterium sibiricum *strain 255-15, growth occurs from -5°C to 40°C. This member of the *Bacillaceae *is Gram-positive, rod-shaped, facultative aerobic, and motile with peritrichous flagella [16]. The relevance of studying this microorganism is that this genus is adapted to diverse cold environments and has been shown to be prevalent in the Siberian permafrost [17-20]. Furthermore, this genus can be found in geological layers frozen for 20 thousands to up to 3 million years, indicating that this genus can endure long freezing periods [17]. This microorganism also grows over an unusually broad temperature range and hence provides a good model for exploring molecular mechanisms of thermal adaptation.

The genome of *E. sibiricum *strain 255-15 has been sequenced to completion by DOE's Joint Genome Institute. The genome has been assembled into three contigs: one chromosome (3034146 bp, 3007 CDS) and two plasmids (1765 bp, 3 CDS and 4885 bp, 5 CDS). This study shows that few transcriptional changes were observed when the microbes were grown at 10°C and 28°C, but several stress related gene expression changes were observed after growth at -2.5°C and 39°C, indicating that *E. sibiricum *strain 255-15 is adapted for growth at cold to moderate range of temperatures.

## Results and discussion

### Genome analysis

*E. sibiricum *CDS have most top hits in BLASTP with species belonging to the same order, *Bacillales *(Table 1). Its GC content was more similar to *Bacillus *species. The *E. sibiricum *genome is approximately the same size as *Listeria innocua *(3.0 Mb). The genome size and the GC content of *E. sibiricum *were also similar to *Psychrobacter arcticus *273-4, a Gram-negative microorganism also isolated from the Siberian permafrost.

**Table 1:** Organisms with similarity to the greatest number of *Exiguobacterium sibiricum *proteins


**Organism**	**Number of Best Hits**	**Genome size**	**% GC content**

*Bacillus halodurans*	429	4.2 Mb	43.7%
*Bacillus subtilis*	379	4.2 Mb	43.5%
*Oceanobacillus iheyensis*	210	3.6 Mb	35.7%
*Listeria innocua*	65	3.0 Mb	37.4%
*Psychrobacter arcticus *273-4	n.d.	2.7 Mb	42.8%

n.d. = not detected in the comparison of *E. sibiricum *CDS with the nr NCBI database.

The cluster of orthologous groups (COGs) distribution, described in the IMG website of *E. sibiricum *genome, shows that approximately 27.7% of the genes do not have a function predicted. Since cold and heat stress related genes are not yet completely elucidated, it is possible that among these large numbers of poorly characterized genes are ones important for the cell acclimation to stress temperatures. The genome also encodes apparent homologs of stress-related proteins as well as many novel proteins that may have unique roles in adaptation to the permafrost environment (Table 2). We analyzed the genome of *E. sibiricum *255-15 according to the following seven categories.

**Table 2:** Known cold and heat stress response proteins with homologs in *Exiguobacterium sibiricum *255-15 listed by categories

**Categories**	**Heat stress**	**Cold stress**	**Cold and heat stresses**
Translation factors	GrpE, DnaJ -chaperone	CspA, C, R, L, RpsF, RbfA, Ef-Tu IF2, IF3, Dead-box RNA helicase, Trigger factor-chaperone	GroEL, GroES, DnaK
Sigma factors	RpoD		RpoE, RpoH
DNA replication	GyrB		GyrA
Membrane alteration		Desaturase, β-ketoacyl carrier protein	
Metabolism		Isocitrate dehydrogenase, Cysteine synthase, Glyceraldehyde phosphate dehydrogenase, Triose phosphate isomerase, Pyruvate dehydrogenase, γ-glutamyltranspeptidase, Dihydrolipoamide acetyltransferase	
Miscellaneous	HtrA, HrcA	NusB, RecA, PNPase	β-Lon protease, General stress proteins

#### Carbohydrate metabolism

**Figure 1:**
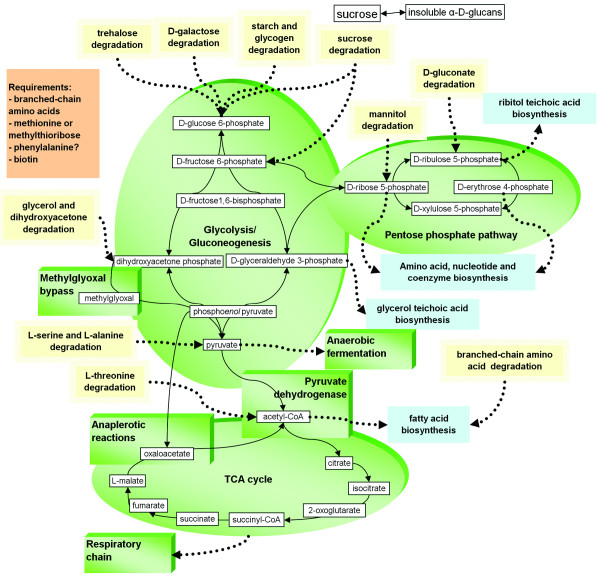
Metabolic pathway reconstruction of *Exiguobacterium sibiricum *255-15 based on genome content.

Genome analysis and previous physiological studies showed that *E. sibiricum *255-15 prefers sugars and carbohydrate polymers as carbon sources [16,21]. All the genes for Embden-Meyerhoff pathway (glycolysis) such as glucose-specific PTS (phosphotransferase), glucose-6-phosphate isomerase, 6-phosphofructokinase, fructose-bisphosphate aldolase, among others, are present (Figure 1). In addition to glycolysis, *E. sibiricum *is capable of gluconeogenesis from glycerol since it has the glycerol utilization operons and fructose-1,6-bisphosphatase [16]. The presence of methylglyoxal synthase is another indicator that *E. sibiricum *prefers glycolytic substrates. This enzyme allows bacteria to bypass the lower part of glycolysis in carbon-rich but phosphorus-limited conditions. *E. sibiricum *has also all the enzymes for the non-oxidative pentose phosphate pathway, but not for its oxidative branch (no glucose-6-phosphate dehydrogenase and phosphogluconolactonase). In support of this analysis, *E. sibiricum *can grow on N-acetylglucosamine, D-ribose, glycerol, dihydroxyacetone, D-glucose, D-gluconate, D-galactose (with two operons), maltose, D-fructose, sucrose, trehalose, and beta-glucosides, such as salicin and arbutin, mannitol (the last five sugars have sugar-specific phosphotransferase systems (PTS)) [16]. The comparison of the *E. sibiricum *PTS genes with other low G+C sequenced genomes showed that among all of them, including *E. sibiricum*, the PTS systems were very distinct, i.e. without any specific pattern of sugar uptake in this group. Karlin et al. [22] suggested that the differences in PTS systems in different genomes are probably due to differences in habitats, lifestyle, and nutrient sources. Genome analysis indicates that *E. sibiricum *may also be able to utilize ethanol and methylthioribose, but its growth with these substrates has not been tested.

**Figure 2:**
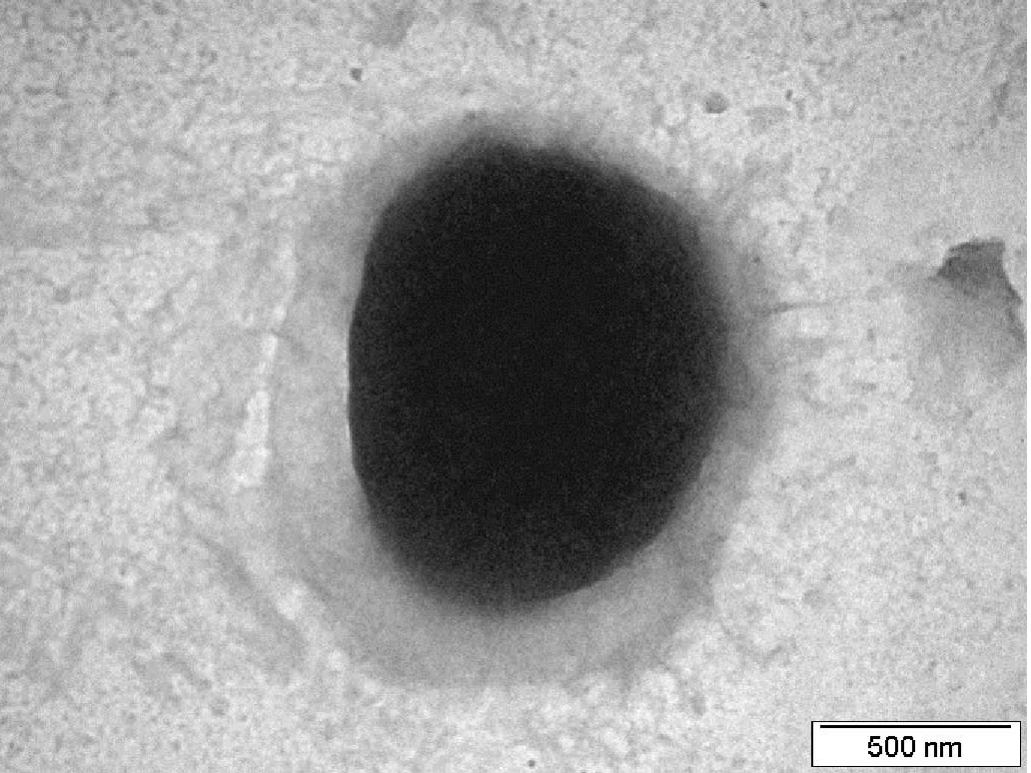
**Negatively stained electron micrograph of *E. sibiricum *strain 255-15 grown at -2.5°C. **Exopolysaccharide and no flagella are observed.

*E. sibiricum *255-15 has a number of enzymes for degradation of carbohydrate polymers, mostly starch and starch-derived oligosaccharides, which were observed in the genome and confirmed experimentally [16]. These include several alpha-amylases, oligo-1,6-glucosidases, non-glucogenic alpha-amylase, exo-alpha-1,4-glucosidase, alpha-glucosidase, pullulanases, and maltose phosphorylase. *E. sibiricum *may also store carbon as a non-branched glucose polymer, similar to glycogen, but without 1 -> 6 branches, since the branching enzyme is absent. The genome analysis showed that *E. sibiricum *can store glucans as extracellular carbohydrate polymers given that it has a putative glucansucrase that synthesizes insoluble alpha-D-glucans from sucrose. Although the linkage pattern of the glucan products cannot be predicted based only on the genome sequence, the specificity of the hydrolases found in the genome suggest 1 -> 4/1 -> 6 alpha-D-glucans as the most likely products. Previous work has shown the presence of granules inside the cell [16] as well as the presence of exopolysaccarides (Figure 2), which could be the result of the synthesis activity of these gene products. The production of exopolysaccharide has also been reported in other cold-adapted microbes: *C. psychrerythraea*, *P. ingrahamii*, and *I. loihiensis*, and their role as cryoprotectants has been suggested [7,8,10].

#### Amino acid biosynthesis

*E. sibiricum *grows on media containing tryptic hydrolysates, which is consistent with its genome-predicted auxotrophy for several amino acids. The enzymes for biosynthesis of branched-chain amino acids (leucine, isoleucine, and valine) and threonine are absent in *E. sibiricum *(Figure 1). It contains a complete pathway for biosynthesis of chorismate, which serves as a precursor of aromatic amino acids. However, only the tryptophan biosynthesis pathway is complete, whereas in phenylalanine and tyrosine pathways only the first enzyme (after chorismate), chorismate mutase, is present, which is fused to 3-deoxy-D-arabino-heptulosonate-7-phosphate (DAHP) synthase. This protein has 65% identity to *B. subtilis *AroA, in which the chorismate mutase domain has relatively low activity and has been hypothesized to serve mainly for feedback regulation and not as a bona fide bifunctional enzyme [23]. Thus, it is likely that *E. sibiricum *is auxotrophic for phenylalanine. Tyrosine, on the other hand, can be produced from phenylalanine by phenylalanine 4-hydroxylase. It is, however, also possible that *E. sibiricum *has an unusual pathway for phenylalanine biosynthesis, since a protein with low similarity to the periplasmic cyclohexadienyl dehydratase of *P. aeruginosa *is present in the genome [24]. The extracellular chorismate-to-phenylalanine pathway similar to the "hidden overflow pathway" of *P. aeruginosa *has never been described in Gram-positive bacteria. Hence, the details of aromatic amino acid biosynthesis in *E. sibiricum *will require experimental clarification.

The genes from the sulfate activation pathway (ATP sulfurylase, APS reductase or APS kinase and PAPS reductase or any of sulfite reductase enzymes) were not found, which indicates that *E. sibiricum *cannot utilize sulfate and requires an organic source of sulfur, such as methionine, cysteine or methylthioribose. *Lactococcus lactis *and *E. sibiricum *have ortholog genes of a putative cysteine (cystine) ABC transporter and a methionine ABC transporter. *E. sibiricum *has a probable serine O-acetyltransferase and cysteine synthase for biosynthesis of cysteine from serine and sulfide. In addition, it seems to have a transsulfurylation pathway from methionine to cysteine represented by cystathionine beta-synthase and cystathionine gamma-lyase. The orthologs of homoserine O-succinyl (O-acetyl) transferase (*metA*) or O-acetylhomoserine sulfhydrylase (*cysD*) were not found in the genome. *E. sibiricum *has cobalamin-independent homocysteine S-methyltransferase and a pathway for methylthioribose-recycling to methionine. Thus, it appears that *E. sibiricum *requires methionine or methylthioribose for growth and can produce cysteine by transsulfurylation from methionine. Lysine biosynthesis appears to be proceeding via acetylated intermediates and employing a dapX-type diaminopimelate epimerase. Conventional amino acid biosynthesis pathways are present for the remaining amino acids including arginine, histidine, glutamate, glutamine, asparagine, serine, and polyamines.

#### Amino acid catabolism

*E. sibiricum *has some amino acid degradation pathways that can be used as energy sources (Figure 1). The operon with NAD-dependent valine dehydrogenase, branched-chain alpha-keto acid dehydrogenase, phosphotransbutyrylase, and butyrate kinase catalyzes oxidative deamination of valine, isoleucine, and leucine. This pathway allows usage of branched-chain amino acids as nitrogen sources and conversion of branched-chain amino acids into the corresponding free acids: isovalerate, isobutyrate, and methylbutyrate. The latter can either be used to generate branched-chain acyl-CoAs for fatty acid biosynthesis or produce ATP through substrate-level phosphorylation under anaerobic conditions.

*E. sibiricum *also has pathways for degradation of the aromatic amino acids phenylalanine and tryptophan. Phenylalanine dehydrogenase is present, which catalyzes the oxidative deamination of phenylalanine to phenylpyruvate, a product that can be further converted to phenylacetate (e.g., by pyruvate dehydrogenase found next to phenylalanine dehydrogenase). Phenylacetate can be further degraded via a ring-hydroxylation/beta-oxidation pathway encoded by an operon next to phenylacetate-CoA ligase. Tryptophan degradation genes encoding tryptophan dioxygenase, kynurenine formamidase and kynureninase are present and could produce enzymes to degrade L-tryptophan to anthranilate. There is no pathway for further degradation of anthranilate, so most likely tryptophan can be used as nitrogen, but not as carbon source.

*E. sibiricum *has a pathway for anaerobic degradation of threonine into glycine and acetyl-CoA via threonine 3-dehydrogenase and 2-amino-3-ketobutyrate-CoA ligase. This pathway allows threonine to be used as nitrogen and probably as carbon source. Glycine can be further degraded via a glycine cleavage system. L-serine dehydratase and alanine dehydrogenase are also present; they produce pyruvate out of L-serine and L-alanine, respectively.

#### Coenzyme and cofactor biosynthesis

*E. sibiricum *is likely auxotrophic for biotin since KAPA synthase, KAPA aminotransferase, dethiobiotin synthase, and biotin synthase are not present in the genome. The biosynthesis pathways of the other known coenzymes are complete and essentially similar to those found in *B. subtilis*.

#### Nucleotide biosynthesis

Both purine and pyrimidine biosynthesis pathways are complete (Figure 1). *E. sibiricum *has both aerobic and anaerobic ribonucleoside diphosphate/triphosphate reductases.

#### Energy metabolism

*E. sibiricum *grows both aerobically and anaerobically [16]. For aerobic growth, the genome contains a complete TCA cycle and a branched aerobic respiratory chain, which consists of monomeric NADH-quinone oxidoreductase, menaquinol-cytochrome c reductase and three terminal oxidases. In some of the low G+C Gram-positive genomes the presence of TCA cycle is only observed in *B. subtilis *and *B. halodurans*, whereas the TCA pathway is entirely missing from both *Streptococcus pyogenes *and *Streptococcus pneumoniae *genomes, and is incomplete in *L. monocytognes*, *L. innocua*, *Clostridium acetobutylicum*, and *Clostridium perfringens *[22]. One terminal oxidase, cytochrome bd-dependent enzyme similar to *B. subtilis *YthAB is likely a quinol oxidase, while two other enzymes, cytochrome caa3 and cytochrome ba3 are cytochrome c oxidases. Although there is no anaerobic respiratory chain observed in the genome, anaerobic growth has been shown to occur via fermentation of sugars [16]. Anaerobic fermentation of branched-chain amino acids and threonine is also possible in *E. sibiricum*. Fermentation pathways found in the genome include pyruvate-formate lyase and acetoin (butanediol). The pyruvate-formate lyase, an enzyme critical in mixed acid fermentation, is present in many enteric γ-proteobacteria and several low G+C Gram-positive sequenced genomes, such as *Lactococcus lactis*, *S. pyogenes*, *S. pneumoniae*, *L. monocytogenes*, *L. innocua*, *Staphylococcus aureus*, and *C. perfringens *[22]. This pathway, however, is missing in *B. subtilis *and *B. halodurans *[22].

#### Miscellaneous observations

*E. sibiricum *has genes that encode the production of both glycerol teichoic and ribitol teichoic acids. It has only one fatty acid desaturase, no fatty acid hydroxylases, and no cyclopropane-fatty acid synthase, but it seems to produce mostly branched-chain fatty acids [21]. It might also be capable of producing rhodopsin and one or more different carotenoids (*E. sibiricum *has two squalene/phytoene synthases, one of them is clustered with a putative diapophytoene desaturase and the other with a phytoene dehydrogenase). The orange pigmentation, observed in *Exiguobacterium *colonies [16], suggests that it does make carotenoid compounds.

The most abundant family of transcriptional regulators in *E. sibiricum *is the MarR family [see Additional file 1: Table S1]. *E. sibiricum *has one unexpected response regulator, *spo*0A, which in *Bacillus *works as a master regulator of sporulation and controls more than 100 genes. Exig_0912 is an ortholog of *spo*0A with 46% identity to *B. subtilis *protein. Orthologs of some genes in the phosphorelay cascade activating *spo*0A (*kin*A, *kin*B, *spo*0B) are also present. A homolog of Spo0F seems to be present as well, with only 38% identity to *B. subtilis *protein. Orthologs of the classic *B. subtilis *sporulation operons under the control of *spo*0A (*spoIIA *and *spoIIG*) are not present in *E. sibiricum*, so most likely it does not sporulate.

This microorganism has several genes that may be involved in thermal adaptation, e.g. heat and cold-shock. The genes listed in Table 2 are present in *E. sibiricum*'s genome and hence may be expressed under temperature stresses. These include several ribosomal binding proteins involved in translation, genes responsible for maintaining the membrane homeoviscous adaptation, sigma factors, genes involved in DNA replication, and genes involved in *E. sibiricum*'s metabolism that may have special temperature adaptation roles. Since the genome analysis showed several stress related genes, we integrated the genome analysis with *E. sibiricum*'s known physiology and its transcriptome responses.

### Transcriptome analysis

**Figure 3:**
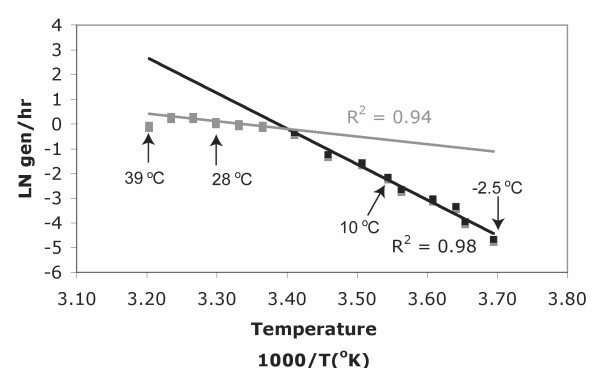
**Arrhenius plot of *E. sibiricum *255-15 growth rates in 1/2 TSB.** The first phase of the biphasic response is in gray and the second phase is in black, each with its respective trend lines and R^2 ^values.

**Figure 4:**
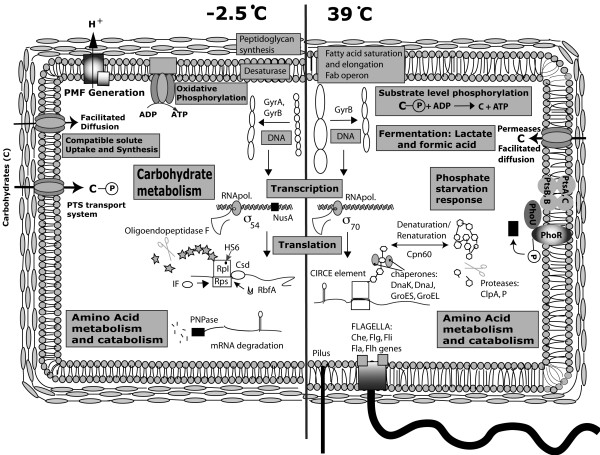
**Comprehensive schematic thermal adaptation of *E. sibiricum *strain 255-15 gene expression results at its extreme growth temperatures. **The left part of the figure summarizes the gene expression results of *E. sibiricum *grown at -2.5°C, when compared to 28°C, 10°C, and 39°C; and the right side does the same for *E. sibiricum *grown at 39°C compared to the other temperatures.

To gain further insight into which of the organism's genes may be involved in temperature adaptations, transcriptome analyses were performed with cells grown at -2.5°C, 10°C, 28°C, and 39°C. The 28°C and 10°C temperatures were chosen as the mean of the maximum and the minimum temperature where the biphasic shift in the growth rate occurred (Figure 3). The temperature of 39°C was selected because it is near the organisms upper limit of growth (40°C) and -2.5°C was selected as a subzero temperature where the medium does not freeze. We also integrated the following interpretations of thermal adaptation into a summary figure of the cell's metabolism at -2.5°C and 39°C (Figure 4).

**Figure 5:**
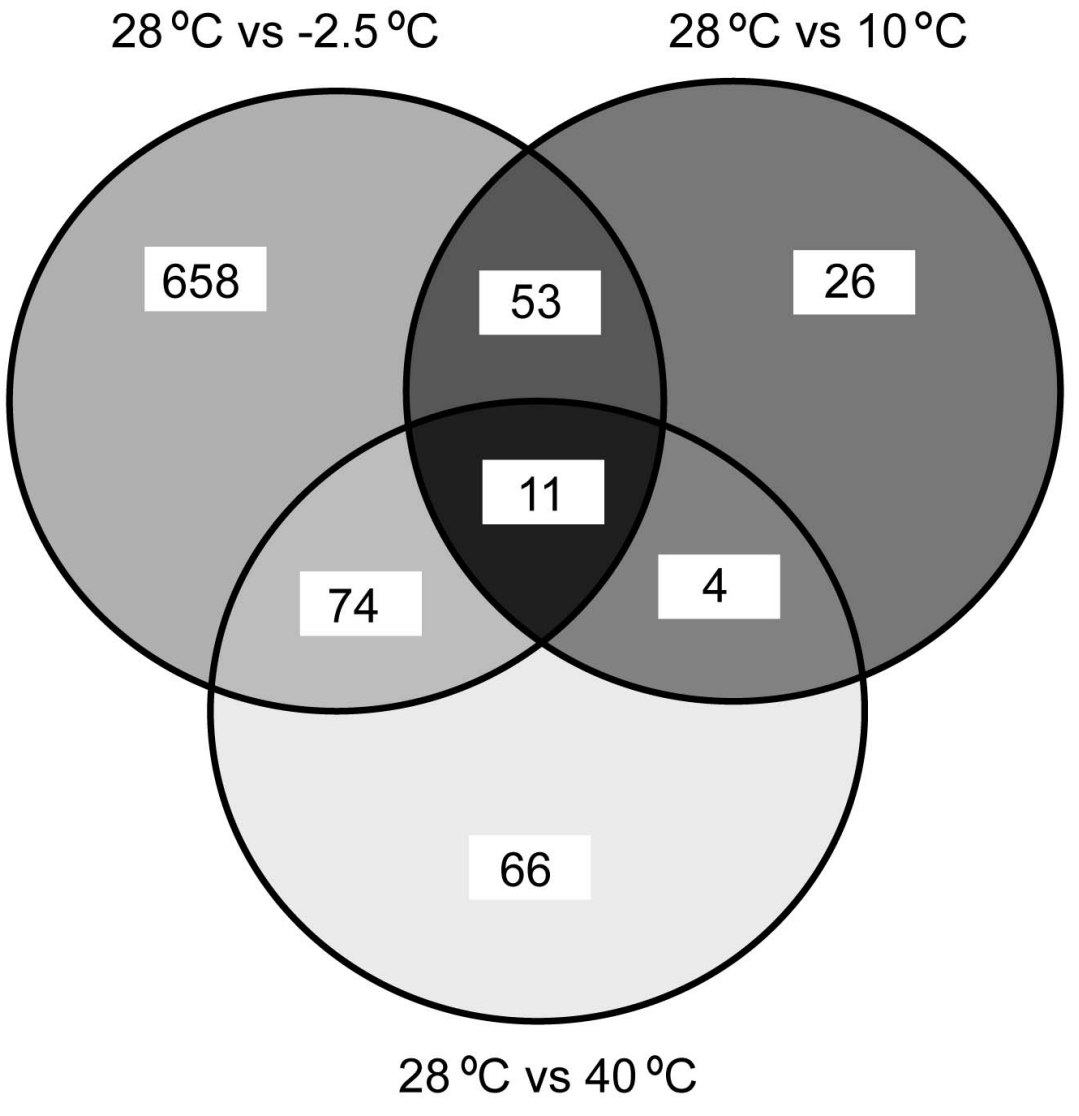
Venn diagram of all differentially expressed genes at the indicated temperatures.

**Figure 6:**
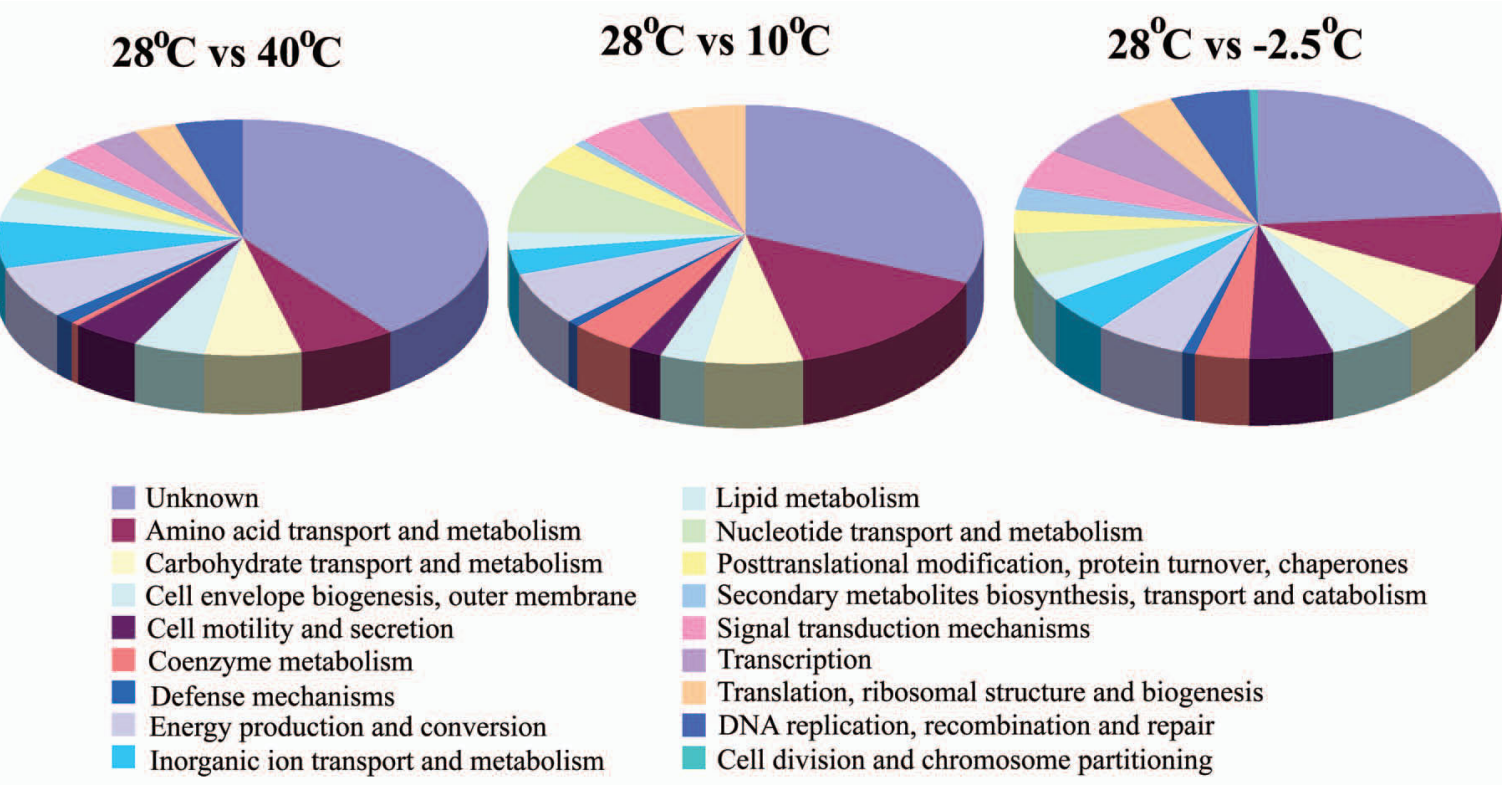
Classification of all genes differentially expressed at the indicated temperatures based on COGs categories.

The overall transcriptome studies showed that 27%, 3.2%, and 5.2% of *E. sibiricum *strain 255-15 CDS spotted on the DNA microarray were differentially expressed in cells grown at -2.5°C, 10°C, and 39°C, respectively, when compared to cells grown at 28°C (Figure 5). The hypothetical genes represented 10.6%, 0.89%, and 2.3% of the CDS differentially expressed when grown at -2.5°C, 10°C, and 39°C versus 28°C, respectively. The genes differentially expressed were clustered based on COGs to classify the genes into main groups (Figure 6). This result showed that many unknown genes were differentially expressed, especially at -2.5°C, suggesting that genes important to thermal adaptation may be in this group. Additionally, genes from the DNA replication, recombination and repair category were differentially expressed at -2.5°C and 40°C when compared to 28°C, while the genes from the cell division and chromosome partitioning category were only differentially expressed at -2.5°C (Figure 6 and see Additional file 1: Tables S2 and S3).

Changes in gene expression commonly observed in cells enduring heat and cold shock were observed in *E. sibiricum *during growth at the upper temperature limit and at subzero temperature, respectively. These changes were in transcripts associated with carbohydrate metabolism, energy metabolism, amino acid biosynthesis and catabolism, membrane and cell wall adaptation, as well as DNA replication, transcription, and translation (Figure 4).

#### Carbohydrate metabolism

*E. sibiricum *seems to have different carbon source preferences related to growth temperatures [see Additional file 1: Table S2]. For instance, *E. sibiricum *can grow on glycerol at 24°C but not at 4°C [21]. In the transcriptome analysis, the genes from the glycerol metabolism, such as the glycerol 3-phosphate dehydrogenase (*glpA*), was up regulated at 39°C, but down regulated at 10°C. At -2.5°C, however, the pathway for glycerol degradation was again up regulated and its differential gene expression was higher than at all the other temperatures (*glpKFA*). The same pattern was seen for D-galactose catabolism genes. Temperature also seemed to affect which carbon compounds were taken up by the cell since we observed up-regulation of all the genes involved in the PTS transport system of glucose at -2.5°C, 10°C, and 28°C when compared to 39°C. The PTS system was down regulated at 39°C but glucose still appeared to be taken up by up-regulated glucose permeases, as was the case for other sugars such as maltose, melobiose, and polymers, since maltose-binding proteins, Na^+^/melobiose transporter, and alpha-amylases were up regulated at 39°C. The fact that enzyme structure and function can be affected by, either low or high temperatures, suggests that cells will increase the synthesis of proteins to compensate for a decrease in activity and stability [25], but in this case the cell may be coping with the different temperatures by changing its carbon source utilization or uptake mechanism.

**Figure 7:**
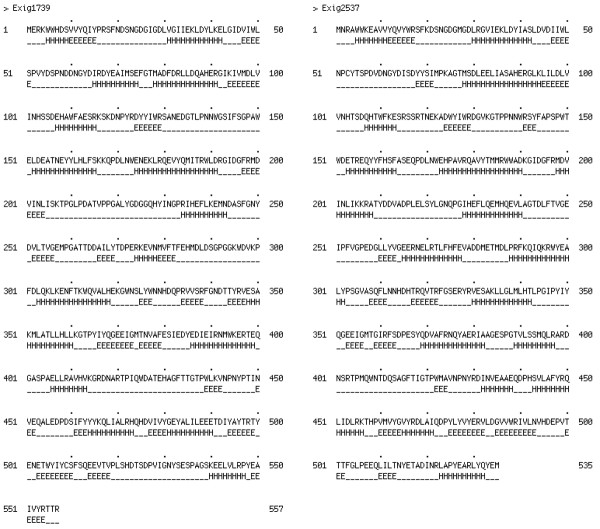
**Secondary structure prediction of two alpha-amylases (Exig_1739 and Exig_2537).** E = extended strand and H = alpha-helix.

Another interesting observation was that several glucosidases (alpha-amylases) were differentially expressed at different temperatures; some were highly expressed and others were down regulated at -2.5°C. In a few cases, we observed gene expression for this type of enzyme to gradually change with temperature. For instance, the alpha-amylase encoded by the gene Exig_1739 was up regulated at -2.5°C, while Exig_2537 was down regulated at -2.5°C and slightly up-regulated at 10°C and 40°C when compared to 28°C [see Additional file 1: Table S2]. The analysis of these proteins showed that Exig_1739 has 22 amino acid residues more than Exig_2537 (Table 3). The secondary structure prediction of Exig_1739 showed that it has additional alpha-helices and extended strands when compared to Exig_2637 (Figure 7). At the amino acid level, Exig_1739 has less arginine residues (27) and more glycine (37 residues), lysine (25 residues), and isoleucine residues (35) than Exig_2537 (35, 32, 15, 27 residues, respectively). These protein features are some of the characteristic changes found in cold-active enzymes relative to their mesophilic and thermophilic counterparts in alpha-amylases and in other cold-adapted proteins [26,27]. These findings suggest that *Exiguobacterium *has alleles (isozymes) for both mesophilic and psychrophilic alpha-amylases, which would explain the preferential gene expression at certain temperatures. Furthermore, we determined by calculating the chi-square that the codon usage for both alpha-amylases were not statistically different (p-value < 0.001) from the *E. sibiricum *genome (Table 4). This result suggests that none of these alleles originated from lateral gene transfer, but they are part of *E. sibiricum*'s physiological adaptation to cope with different temperatures.

**Table 3:** Properties of alpha-amylase proteins: Exig_1739 and Exig_2537

**Properties**	**Exig_1739**	**Exig_2537**
Number of residues	557	535
Charge	-35.0	-23
Tiny (A+C+G+S+T)	129	124
Small (A+B+C+D+G+N+P+S+T+V)	265	252
Aliphatic (A+I+L+V)	137	140
Aromatic (F+H+W+Y)	86	83
Non-polar (A+C+F+G+I+L+M+P+V+W+Y)	286	287
Polar (D+E+H+K+N+Q+R+S+T+Z)	271	248
Charged (B+D+E+H+K+R+Z)	163	144
Basic (H+K+R)	68	64
Acidic (B+D+E+Z)	95	80

**Table 4:** Codon usages and amino acid frequencies per 1000 of *E. sibiricum *255-15 genome and alpha-amylase proteins (Exig_1739 and Exig_2537)

**Codon**	**a.a.**	**255-15**	**1739**	**2537**			**255-15**	**1739**	**2537**			**255-15**	**1739**	**2537**			**255-15**	**1739**	**2537**
GCA	A	22.25	7.17	7.45	CAC	H	8.34	14.34	9.31	CCA	P	6.40	12.55	1.86	TCG	S	14.80	10.75	13.04
GCC	A	22.34	17.92	20.48	CAT	H	13.16	14.34	16.76	CCC	P	2.84	7.17	5.59	TCT	S	5.67	7.17	1.86
GCG	A	23.65	16.13	27.93	ATA	I	2.11	0.00	1.86	CCG	P	24.40	26.88	40.97	ACA	T	19.13	16.13	7.45
GCT	A	12.94	10.75	5.59	ATC	I	36.04	32.26	16.76	CCT	P	4.30	3.58	5.59	ACC	T	10.08	3.58	9.31
																			
TGC	C	1.99	1.79	0.00	ATT	I	28.88	30.47	31.66	CAA	Q	24.76	14.34	20.48	ACG	T	29.17	30.47	33.52
TGT	C	3.84	0.00	1.86	AAA	K	39.13	35.84	18.62	CAG	Q	17.85	14.34	22.35	ACT	T	4.76	7.17	3.72
GAC	D	20.69	34.05	27.93	AAG	K	14.14	8.96	9.31	AGA	R	1.97	5.38	0.00	GTA	V	7.26	10.75	5.59
GAT	D	32.71	50.18	48.42	CTA	L	3.05	1.79	1.86	AGG	R	0.59	0.00	1.86	GTC	V	41.07	26.88	42.83
																			
GAA	E	53.26	64.52	44.69	CTC	L	19.68	7.17	5.59	CGA	R	6.12	5.38	9.31	GTG	V	10.37	5.38	9.31
GAG	E	17.44	21.51	27.93	CTG	L	22.47	21.51	27.93	CGC	R	9.79	8.96	11.17	GTT	V	14.77	12.55	9.31
TTC	F	22.14	14.34	14.90	CTT	L	16.08	8.96	3.72	CGG	R	17.76	17.92	31.66	TGG	W	9.70	30.47	31.66
TTT	F	21.48	17.92	20.48	TTA	L	20.34	17.92	20.48	CGT	R	16.41	10.75	11.17	TAC	Y	13.81	25.09	26.07
																			
GGA	G	22.99	26.88	22.35	TTG	L	22.53	17.92	22.35	AGC	S	6.96	5.38	5.59	TAT	Y	18.94	37.63	35.38
GGC	G	14.35	5.38	13.04	ATG	M	25.80	23.30	31.66	AGT	S	9.57	10.75	9.31	TAA	*	2.02	1.79	1.86
GGG	G	10.23	16.13	13.04	AAC	N	16.91	26.88	18.62	TCA	S	10.86	10.75	9.31	TAG	*	0.13	0.00	0.00
GGT	G	21.50	17.92	11.17	AAT	N	15.59	26.88	24.21	TCC	S	8.36	8.96	14.90	TGA	*	1.36	0.00	0.00

The Chi-square results for the codon usage were not statistically significant for any of the two proteins compared to the genome.

a.a. = amino acid

In the genome analysis we showed that *E. sibiricum *can produce intra and extracellular polymers composed by carbohydrates. Storage granules were observed previously by transmission electron microscopy [16], as was an external capsule (Figure 2). In the transcriptome analysis several genes related to capsular polysaccharide biosynthesis were also expressed. Although a number of genes coding for exopolysaccharide synthesis were down regulated at -2.5°C, when compared to 10°C, microscopic observations showed that the exopolysaccharide was present at all temperatures, including at -2.5°C (Figure 2). The reduction in gene expression levels can be explained by the substantially slowed metabolism of *E. sibiricum *at -2.5°C, but still sufficient to produce the observed exopolysaccharide.

#### Energy metabolism

Further insight into the observed anaerobic growth of *E. sibiricum *came from the transcriptome analysis at 39°C (Figure 4 and see Additional file 1: Tables S2 and S3). At this temperature several genes from different pyruvate fermentation pathways were expressed, such as pyruvate: ferrodoxin oxidoreductase, pyruvate-formate lyase, and L-lactate dehydrogenase. This can be explained by the low oxygen solubility at 39°C, about 6.1 mg l^-1 ^[28,29], an amount close to the survival limit of most vertebrates [29]. Additionally, the higher culture medium viscosity and the organism's high metabolic rate at this temperature would further decrease the oxygen availability and likely trigger the expression of fermentation pathways.

Another indication that *E. sibiricum *changes its cell bioenergetics at the temperature extremes is the fact that ATPase sythase and cytochrome synthesis (*cyoCBAE*) operons were down regulated at -2.5°C and 39°C (Figure 4). These two operons are used for ATP synthesis and generation of proton motive force during aerobic respiration. However, the reasons for down regulating these genes are likely different for these extreme temperatures. In the case of -2.5°C, the cells are probably obtaining energy by proton motive force (PMF) since O_2 _is highly soluble at this temperature and no expression of genes related to substrate-level phosphorylation were detected. The lower expression of PMF genes at -2.5°C, when compared to 10°C and 28°C, are probably due to the low metabolic rate at this temperature. On the other hand, the low expression of ATP synthase and cytochrome c genes at 39°C is probably related to decreased respiration, i.e. PMF, caused by the low O_2 _concentration in the medium. This is consistent with a switch from oxidative phosphorylation to substrate-level phosphorylation indicated by the expression change of two genes known to be expressed in microorganisms under anaerobic conditions: pyruvate-formate lyase (*pfyD*) and pyruvate-ferrodoxin oxidorectase (*porA*) [30].

At 39°C all the genes for inorganic phosphate starvation response [30] are up regulated (*pstABC *and *phoU*, Figure 4), however, it is not clear why high temperature could affect phosphate availability to the cell.

#### Amino acid biosynthesis and catabolism

Amino acids in the cells are not only important as building blocks and energy sources but also as osmoprotectants [11]. The uptake and accumulation of compatible solutes, such as glycine betaine and carnatine in cold-stressed cells are known adaptive responses to low temperatures of many microorganisms [31-33]. At -2.5°C and 10°C the transport system of osmoprotectants, such as glycine betaine, carnitine, and choline, were up regulated and completely down regulated at 39°C (Figure 4 and see Additional file 1: Table S2). Additionally, proline dehydrogenase, which is responsible for the first step of proline conversion to glutamate, was progressively up regulated with decreasing temperatures, reaching the highest gene expression at -2.5°C. In *E. coli *the shift to higher osmolarity triggers the accumulation of glutamate as an osmoprotectant [34]. At -2.5°C the medium becomes supercooled [35], which generates water flow out of the cell similar to salt stress response. Hence, the decrease in temperature may be sensed by the cell as a different chemical potential triggering the production of glutamate from proline degradation as well as the uptake of other osmoprotectants. In addition, genes for carnitine degradation, which generate glycine betaine as an osmoprotectant, were also upregulated at -2.5°C. Therefore, besides the cold stress *per se*, cold temperatures also seemed to affect the cell's osmotic homeostasis.

*E. sibiricum *also seems to be changing its amino acids metabolism, especially at -2.5°C. Histidine, serine, arginine, and lysine biosynthesis genes were up regulated at -2.5°C when compared to the other temperatures. Hence, cold stress seems to lead to flux and pool size redistribution throughout the entire network of amino acid metabolism. A similar effect was observed in cowpea cells under heat shock, which modified their metabolism and concentrations of diverse amino acids in the cells [36]. The reason for a change in cell metabolism is uncertain, but it may be for synthesizing more flexible proteins for function at lower temperatures.

#### Cell membrane and cell wall adaptation

It is well known that after cold shock, bacterial cells modify their membranes by increasing unsaturation in the membrane phospholipids and decreasing chain length of fatty acids [37-40] to help maintain the homeoviscosity and hence, function [39-41]. This phenomenon has been extensively studied in *Anabaena variabilis *and *Synechocystis *PCC 6803. Mutants defective in the desaturation of fatty acids (*des*A) have a lower growth rate at low temperatures [42]. Furthermore, *Vibrio sp*. and *Micrococcus cryophilus *adapt to low temperatures by decreasing the chain length of their membrane fatty acids [38,39]. Further studies of cold-shock show an increased degree of unsaturation in the cold-adapted lipid A of *E. coli *[43] and changes in the branched fatty acid profile of *B. subtilis*[44].

*E. sibiricum *also seems to change its saturation and chain length under different temperature conditions (Figure 4). Previous study demonstrated that *E. sibiricum *255-15 shifted from saturated to unsaturated fatty acids at 4°C, consistent with our finding of an increase in fatty acid desaturase gene expression at 10°C and -2.5°C [21]. The fatty acid C16:0 was the predominant fatty acid in *E. sibiricum *255-15 at the mesophilic temperature but at 4°C a shift to iso C17:0 occurred [21]. Therefore, *E. sibiricum *seems to be keeping the membrane homeoviscosity at low temperatures by increasing unsaturation of its fatty acids.

Another noticeable change in *E. sibiricum *255-15 that occurred only at -2.5°C was the increase in expression of peptidoglycan biosynthesis genes (*murADEI*) as well as lysine biosynthesis genes (*dapABD*), which is one of the main amino acids in the *E. sibiricum *peptidoglycan structure [16] [see Additional file 1: Table S2]. A thickening of the cell wall at -2.5°C was observed by an increased difficulty in lysing the cell with lyzozyme during the RNA extraction. This thickening of the cell wall may protect the cell against disruption by ice formation and/or osmotic pressure that can be generated at subzero temperatures.

#### DNA replication, transcription, and translation dynamics

Under cold conditions, DNA becomes more negatively supercoiled [40,41], while under heat stress the DNA becomes less negative supercoiled [45]. In both cases, the DNA must be stabilized in a more functional conformation. In the case of cold stress, nucleoid-associated proteins such as Gyrase A, IHF, and H-NS are suggested to be necessary for its relaxation [46-48]; while for heat stress, only gyrase A has been described as being important. In *E. sibiricum*, only at -2.5°C and 39°C DNA topoisomerases were up-regulated (gyrase B for both temperatures and gyrase A only at -2.5°C DNA), suggesting that *E. sibiricum *uses its DNA topoisomerases to adapt its DNA supercoiling to the temperature extremes [see Additional file 1: Table S2].

We observed several genes encoding transcriptional regulators in *E. sibiricum*, including ARO8, MarR, and GntR family proteins [see Additional file 1: Table S1], which showed significant expression changes at 39°C. The MarR family transcriptional regulators were also induced in *B. subtilis *and *Thermothoga maritima *during heat stress response [49,50]. In the case of *T. maritima *[50], the expression changes of MarR family proteins were dramatically higher during a long-term heat adaptation experiment, which were similar to the responses we observed in *E. sibiricum *at 39°C [see Additional file 1: Table S2]. This suggests that the MarR family proteins may play a role in the cell's adaptation under high temperatures.

At cold temperatures, for instance, the transcription factor NusA is known to be involved in both termination and antitermination of transcripts in *E. coli*, and is highly expressed under low temperatures [38,51]. We also found this gene up regulated at -2.5°C and 10°C in *E. sibiricum*. Additionally, the RNA polymerase sigma 54 (*rpoN*) was up regulated and the RNA polymerase sigma 70 (housekeeping sigma) was down regulated only at -2.5°C, suggesting that sigma 54 may be responsible for the transcription of genes under cold or subzero conditions [see Additional file 1: Table S2].

Besides transcription, cells need to cope with the translation of the transcripts produced, and for that the cells need to have all ribosome-associated proteins required for the formation of the translation complex. During cold-shock, several studies demonstrated that the genes favoring the formation of the translation complex at cold temperatures are fully functional, such as DeaD-box RNA helicase, ribosome binding factor A (*rbfA*), initiation factor 2 (IF-2) [38,41,52] among several other ribosomal proteins. RNA helicases were up-regulated in *E. sibiricum *at -2.5°C and 10°C, which may help unwind the RNA secondary structure for efficient translation at low temperatures [38,53]. However, it was only at -2.5°C that IF-2, IF-3, and *rbfA *were significantly up regulated in *E. sibiricum *[see Additional file 1: Table S2]. Different temperatures, especially at -2.5°C and 39°C, also affected the transcription of ribosomal proteins. The transcript of the ribosomal protein L25 (*rplY*) was up regulated at 39°C and -2.5°C; while Rpl8A (L7Ae protein family) was up regulated only at -2.5°C. On the other hand, the ribosomal protein transcript of *rpmF *was down regulated at -2.5°C [see Additional file 1: Table S2]. This change in ribosomal protein expression in the cold has also been observed in several other microorganisms, e.g. *Lactobacillus plantarum *[54], *Halobacterium *sp. NRC-1 [55], and *B. subtilis *[56], among others. Since these proteins seem to be expressed under cold conditions in different microorganisms and are part of the ribosomal proteins, they probably play an important role in the translation complex at cold temperatures.

After the mRNA is transcribed and translated it needs to be degraded. It has been shown that during cold-shock, the RNA produced is degraded by a 'cold-adapted' RNA degradosome that contains PNPase (polyribonucleotide nucleotidyltransferase) [41,46]. *E. sibiricum *also increased its *pnp *gene expression at -2.5°C, which suggests that *E. sibiricum *may need this gene product for growth in cold temperatures [see Additional file 1: Table S2].

The proteins that come out of the translational machinery need to be folded properly to be fully functional. At high temperatures this can be a problem since protein denaturation can occur. *E. sibiricum *seems to overcome this problem at 39°C by inducing genes of diverse heat shock proteins. These proteins are homologous to *B. subtilis *and *T. maritima *class I heat-shock genes *hrcA-gprE-dnaJ-dnaK *and *groEL-groES*. In *E. sibiricum *255-15, two operons are observed in the reverse strand: the first contains the genes *dnaK, grpE *and *hrcA*; the other operon contains *adk *(Cpn60) and *groL*. Both operons presented similar expression levels at 39°C. The gene for *dnaJ *was also highly expressed at 39°C, but it is not part of the same operon that contains *dnaK*, as seen in *B. subtilis *[57]. Additionally, some genes encoding ATP-dependent proteases *clpA *and *clpP *were also up regulated in *E. coli *and *B. subtilis *under heat stress [50,58,59], as well as in *E. sibiricum *at 39°C. Futhermore, the genes *dnaK*, *groES*, *cpn60 *were downregulated at -2.5°C and their expression increased with an increase in temperature and became highly expressed at 39°C [see Additional file 1: Table S2].

#### Miscellaneous observations

**Figure 8:**
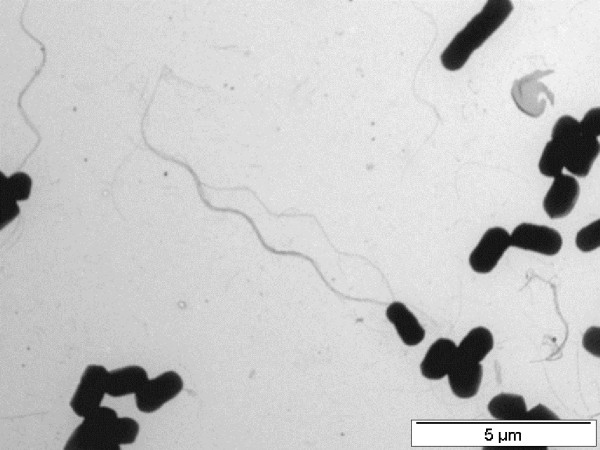
**Negatively stained electron micrograph of *E. sibiricum *strain 255-15 grown at 10°C. **Flagella are observed.

Even though at 10°C *E. sibiricum *has flagella (Figure 8), at -2.5°C the flagella is absent (Figure 2). Furthermore, at -2.5°C all the *che *genes in the operon, as well as all the genes involved in flagella synthesis (all the genes from *flh*, *fli *and *flg *operons), were down regulated [see Additional file 1: Table S2]. Subzero temperatures can make the medium more viscous or frozen, making flagella useless. Additionally, several genes for pilus assembly proteins (*pil *genes) and type II secretory pathway (*pul *genes) were also down regulated at -2.5°C.

## Conclusion

In conclusion, few differences in gene expression related to cold adaptation were observed in *E. sibiricum *at the temperatures 10°C and 28°C. Similar results were obtained at 25°C and 4°C in proteome profiles of *E. sibiricum *[60]. This work demonstrates that this microorganism is constitutively adapted to cold temperatures since at stressful temperatures for mesophiles, such as 4°C and 10°C, genes related to temperature stress are not differentially expressed. Even though the growth rates change between 10°C and 28°C, it was surprising to see very little difference in gene expression and no expression of genes related to stress, especially because these two temperatures belong to two different phases of the biphasic Arrhenius plot (Figure 3). It seems that the growth rate shift in the Arrhenius plot does not have a strong significance on the microorganism physiology, at least observable at the transcriptome level by microarray technology. On the other hand, at the extremities of the Arrhenius profile, *E. sibiricum *undergoes several physiological adaptations very similar to cold and heat shock responses previously studied in other organisms. These physiological adaptations in *E. sibiricum *involved: different carbon source utilization at different temperatures or the presence of mesophilic and psychrophilic alleles of the same protein expressed at specific temperatures; switching energy metabolism from oxidative phosphorylation to substrate-level phosphorylation at 39°C; changes in amino acid metabolism by producing diverse osmoprotectants to maintain the osmotic homeostasis at colder temperatures, but also by increasing the production of certain amino acids at different temperatures; cell membrane and cell wall adaptation by changing the saturation and chain length of the membrane fatty acid and by thickening the peptidoglycan at -2.5°C; changes in transcription and translation machinery by expressing diverse transcriptional regulators and other important proteins to ensure cell functionality. Previous studies suggested that these physiological or gene/protein expression changes were only temporary during the initial shock [11,40], while this study demonstrates that some of the changes in gene expression are not transient but presumably necessary in the longer term for the cell survival and growth at these more extreme growth temperatures.

## Methods

### Bacterial strain and genome sequence

*E. sibiricum *255-15 was isolated from a depth of 43.6 m in the permafrost sediment of the Kolyma Indigirka Lowland [16]. At this depth, sediments are -10 to -12°C and are estimated to have been continuously frozen for 2 to 3 million years. The complete genome sequence was produced by the Department of Energy's Joint Genome Institute and annotated by the automated pipeline operated by Oak Ridge National Laboratory's Computational Genomics Group. The annotation used in this work is available at JGI/ORNL dated December 20th, 2007 . The genome sequence is available online at NCBI [GenBank: CP001022 to CP001024]. The strain has been deposited in 3 culture collections under the accessions: DSM 17290, JCM 13490, and VKM B-2376.

### Arrhenius profile

*E. sibiricum *strain 255-15 was grown in flasks containing 1/2 Tryptic Soy Broth (TSB) (Difco, Detroit, MI) shaken at 200 rpm, at temperatures from 39°C to -2.5°C (precision +/- 0.2°C). Growth rates were calculated from the slope of optical densities for four or more time points for each temperature and summarized as an Arrhenius relationship.

### Transmission electron microscopy

Inoculated agar plates were incubated at 30°C, 12°C, and -2.5°C, until they reached a colony size of 1 to 2 mm of diameter. Cell morphology and flagella were examined by transmission electron microscopy (TEM).

### Probe design and array construction

The genome of *Exiguobacterium *strain 255-15 was used to select gene-specific or group-specific oligonucleotide (70 mer) probes using CommOligo [61] with group-specific probe design features. The design criteria were as follows: (i) 85% sequence similarity, 18-base stretch, and -35 kcal/mol free energy for gene-specific probes; and (ii) 96% sequence similarity, 55-base stretch, and -90 kcal/mol free energy for group-specific probes. Based on those criteria, 2931 CDS had gene-specific probes; 25 CDS were covered by six group-specific probes; no qualified probes were selected for 22 CDS. In addition, 10 human and 10 Arabidopsis probes were designed as controls. Those probes are expected to be very specific since the criteria used were even stricter than those previously suggested [62]. All designed oligonucleotides were commercially synthesized without modification by MWG Biotech Inc. (High Point, NC). The concentration of oligonucleotides was adjusted to 100 pmol μl^-1^. Oligonucleotide probes prepared in 50% DMSO (Sigma Chemical Co., MO) were spotted onto UltraGAPS glass slides (Corning Life Science, NY) using a Microgrid II robotic arrayer (Genomic Solutions Inc., MI). Each oligonucleotide probe had two spots on a single slide. Additionally, six different concentrations (5~300 ng μl^-1^) of genomic DNA were also spotted (four duplicates on a single slide) as positive controls. After printing, the oligonucleotide probes were fixed onto the slide by UV cross-linking (600 mJ of energy) according to the protocol of the manufacturer (Corning Life Science, NY).

### Growth conditions

All the cells for the DNA microarray experiments came from the same *E. sibiricum *255-15 frozen stock that was used for the genome sequencing. All experiments were performed by first plating the cells in 1/2 Tryptic Soy Agar (TSA) and then transferred to 1/2 Tryptic Soy Broth (TSB) twice. In total, six samples were grown independently at 39°C, 28°C, 10°C, and -2.5°C. For the growth at 39°C and 28°C the plates were incubated overnight. At 10°C, the plates were incubated for 3 to 4 days. At -2.5°C, the plates were transferred three times to new plates to acclimate the cells to lower temperature as follows: the first plates were incubated overnight at 22°C followed by incubation at 4°C for 3 days and then for 2–3 weeks at -2.5°C. After growing the cells in agar at the four temperatures, a loop from each plate was transferred to tubes containing 5 ml 1/2 TSB and grown in its respective temperatures until an optical density at 600 nm (OD_600_) of 1.0 was attained. Then 1 ml of this culture was used to inoculate 100 ml of 1/2 TSB in a Nephlo Flask (Belco). The samples were incubated until reaching mid-log growth (0.1 < OD_600 _< 0.3) when 100 ml of RNAlater (Ambion, Austin, Texas) was added at the same temperature as the grown cells. Cells were pelleted by centrifugation at 5,000 × *g *for 20 min at 4°C and resuspended in 1 ml of RNAlater, transferred to a 1.5 ml microcentrifuge tube, and re-pelleted at 5,000 × *g *at 4°C for 10 min.

### RNA isolation

The cells were re-suspended in 100 μl of RNase-free 3 mg ml^-1 ^Lysozyme in TE buffer pH 8 (50 mM Tris-Cl and 1 mM EDTA) by vortexing and then incubated at room temperature for at least 20 min or until the pellet cleared. The RNA was then isolated using the RNeasy mini Prep kit (Qiagen) according to the manufacturer's instructions; the step of DNase digestion was included. The resulting RNA was checked by denaturing agarose gel electrophoresis for DNA contamination and for the presence and integrity of the rRNA bands. The amount of RNA was quantified using a UV-spectrophotometer at OD_260_.

### cDNA labeling and slide hybridization

Amino-allyl labeling was performed as adapted from a protocol of The Institute for Genomic Research (TIGR) . Briefly, 10 μg of total RNA was used to synthesize cDNA overnight at 42°C using 0.5 mM of Random Hexamer Primers (Invitrogen, Carlsbad, CA), 3:2 ratio of 5-(3-amino-allyl)-dUTP and dTTP (Ambion), and Superscript II reverse transcriptase (Invitrogen), and subsequently labeled by coupling reactive Cy5 or Cy3 fluorophores (Amersham, Piscataway, NJ) to the amino-allyl groups. Purification after cDNA synthesis and chemical coupling were performed using QiaQuick PCR purification columns (Qiagen) as described in TIGR protocol. The quantity of labeled cDNA and the fluorophore incorporation efficiency were determined by using UV-visible spectrophotometry.

Microarray slides were incubated for 60 min at 46°C with prehybridization solution (50% Ultrapure formamide (Invitrogen), 5× SSC, 0.1% SDS and 0.1 mg ml^-1^), washed three times in double-distilled water and one time in isopropanol, and dried by centrifugation at 50 × *g *for 3 min. Two cDNA's from different temperatures were mixed for direct comparisons for all temperature combinations. Each microarray received about 30 μl of hybridization solution (50% Ultrapure Formamide, 5× SSC, 0.1% SDS, 0.1 μg μl^-1 ^Salmon sperm DNA) containing the two cDNAs. The solution was applied by capillary action under a coverslip (LifterSlip; Erie Scientific Company, Portsmouth, NH) placed over the microarray. The whole assembly was sealed in a hybridization chamber (CMT Hybridization Chamber; Corning Incorporated, Corning, NY) and submerged for 16 h in a 46°C water bath. Microarray slides were washed twice for 5 min at 46°C with 1× SSC – 0.1% SDS; twice for 10 min at room temperature with 0.1× SSC – 0.1% SDS and five times for 1 min at room temperature with 0.1 × SSC. Slides were dried by centrifugation at 50 × *g *for 3 min and were immediately scanned and analyzed. All microarray data and the 70 mer array v. 1.0 information are available at GEO (Gene Expression Omnibus) [GEO: GSE10133, GEO: GSM256115- GSM25615, GEO: GPL6358].

### Data analysis

Slides were scanned with an Axon 4000B scanner and GenePix 5.0 used for spot finding. Only spots with more than 80% of pixels greater than background plus 2 standard deviations in either Cy5 or Cy3 channel were used for analysis. Analysis was performed with *Limma *(Linear models for microarrays data) library in the CARMAweb environment [63]. The background correction was done by background subtraction of the median value, followed by within and between arrays data normalization using the print tip Lowess method, and quantile method, respectively. A moderated t-test based on empirical Bayes approach (from the Bioconductors *Limma *package) with an adjustment of the calculated raw *P*-values was used with the following methods: Benjamini and Hochberg [64], Westfall and Young [65] as well as Bonferroni. Only *P*-values smaller that 0.01 for all these methods and an expression change higher than 2 folds were considered statistically significant for further analysis.

### Protein and codon usage analyses

For the codon usage analyses of the CDS from the *E. sibiricum *genome and the two alpha-amylases (Exig_1739 and Exig_2537) we used the program CUSP (create a codon usage table) within the European Molecular Biology Open Software Suit (EMBOSS) [66]. The chi-square test comparing the codon usage results of the genome with each alpha-amylase was done with GraphPad Software, Inc. (La Jolla, CA) using 41 degrees of freedom (59 codons that code for 18 amino acids). The start codon and the tryptophan codon were omitted in the analysis because they occur only once, and therefore there is no codon bias. The stop codons were also omitted in the analysis because they occur only once per ORF, but are listed at a higher frequency in the concatenated genome sequences. Thus, the stop codons in the codon bias analysis would artificially inflate the chi-square statistics. The results were considered statistically significant for the calculated chi-square values that were greater than the values in the chi-square table for 41 degrees of freedom with P value of 0.01. For the protein statistics and for the secondary protein structure prediction we used pepstat and predator [67] programs within Mobyle portal [68].

## Authors' contributions

DFR generated all TEM images, analyzed the genome for stress related genes, annotated manually all genes described in this manuscript, designed the microarray experiments, analyzed, interpreted, and linked all the microarray data into pathways, did the protein analyses, and wrote the manuscript. NI analyzed the genome in a holistic view, prepared Figure 1 and additional file 1: Table S1 for this manuscript and made significant contributions to writing the paper. ZH and JZ designed and spotted the oligos into arrays, and revised critically the manuscript. MH gave initial support in the statistical analysis of the microarrays data and revised critically the manuscript. JMT supervised, advised, acquired the funding for this work, and revised critically the manuscript.

## Supplementary Material

Additional file 1 **Table S1 for regulation systems found in *E. sibiricum *genome, and Tables S2 and S3 for the transcriptome comparison results** Click here for file
