# Supplementary material for: Architecture of thermal adaptation in an Exiguobacterium sibiricum strain isolated from 3 million year old permafrost: A genome and transcriptome approach
Source: BMC Genomics. 2008 Nov 18;9:547. doi: 10.1186/1471-2164-9-547 (PMC2615787; doi:10.1186/1471-2164-9-547)
Supplement: Additional file 1 — Table S1 for regulation systems found in E. sibiricum genome, and Tables S2 and S3 for the transcriptome comparison results [file 1471-2164-9-547-S1.doc]

**SUPPLEMENTAL TABLES**

Table S1. Regulation systems found in *E. sibiricum* genome

| **Regulation system** | **Gene number** | **Other characteristics** |
| --- | --- | --- |
| **Histidine Kinases** | | |
| Signal transduction histidine kinase, LytS | Exig_0274, Exig_0228 | LytS/LytR |
| PAS/PAC sensor signal transduction histidine kinase | Exig_2875, Exig_3030,  Exig_1017, Exig_2203 | Walk/WalR, ResE/ResD, PhoR/PhoB |
| Multi-sensor signal transduction histidine kinase | Exig_2756 |  |
| Integral membrane sensor signal transduction histidine kinase | Exig_2665, Exig_1049, Exig_0964, Exig_0732, Exig_0643, Exig_0359, Exig_2930, Exig_2885, Exig_2844, Exig_2691, Exig_2604, Exig_2493, Exig_2450, Exig_2178, Exig_0837, Exig_0724, Exig_0600, Exig_0514, Exig_0588 | CiaH/CiaR, yxdK/yxdJ, cssS/cssR, LiaS/LiaR |
| Histidine kinase | Exig_1216, Exig_2469, Exig_1156 | degS/degU |
| CheA signal transduction histidine kinase | Exig_1857 | CheA |
| **Response regulator receiver domain-containing proteins** | | |
| Two component transcriptional regulator, winged helix family | Exig_2494, Exig_2929, Exig_2451, Exig_2843, Exig_0515, Exig_2692, Exig_0723, Exig_2886, Exig_0644, Exig_2177, Exig_2605, Exig_1016, Exig_2204, Exig_3031 | yxdJ, ciaR?, cssR, resD, phoB, walR |
| Two component transcriptional regulator families | Exig_0227, Exig_0599, Exig_2468, Exig_0589 | lytR, liaR, degU, AraC |
| Sporulation transcriptional activator | Exig_0912 | spo0A |
| Response regulator receiver protein | Exig_1866, Exig_2732, Exig_2142, Exig_0985 | CheY, CheW |
| **Transcriptional regulators** | | |
| Transcriptional regulator, XRE family | Exig_2580, Exig_2526, Exig_1433, Exig_1350 | XRE family |
| Transcriptional regulator, TetR family | Exig_2897, Exig_1440, Exig_2167, Exig_0197, Exig_0183, Exig_0525, Exig_1053 | TetR family |
| Transcriptional regulator, RpiR family | Exig_0468 | RpiR family |
| Transcriptional regulator, PadR family | Exig_0995, Exig_1524, Exig_0305 | PadR family |
| Transcriptional regulator, PaaX family | Exig_0399 | PaaX family |
| Transcriptional regulator, MerR family | Exig_1380, Exig_1082, Exig_0272 | MerR family |
| Transcriptional regulator, MarR family | Exig_2792, Exig_1320, Exig_1222, Exig_1353, Exig_1137, Exig_1360, Exig_1630, Exig_0199, Exig_2969, Exig_1626, Exig_2179, Exig_0366, Exig_2989, Exig_2892, Exig_1356, Exig_0333 | MarR family |
| Transcriptional regulator, LysR family | Exig_0347, Exig_1472, Exig_1654, Exig_1324, Exig_1119, Exig_1327, Exig_2856, Exig_2020, Exig_1673 | LysR family |
| Transcriptional regulator, LacI family | Exig_2894, Exig_2388, Exig_2242, Exig_0321, Exig_0754, Exig_1659 | LacI family |
| Transcriptional regulator, HxlR family | Exig_0155, Exig_2373, Exig_0282, Exig_1935 | HxlR family |
| Transcriptional regulator, GntR family | Exig_1190, Exig_1448, Exig_1824, Exig_0009, Exig_2701, Exig_2215, Exig_1393, Exig_2704, Exig_2995, Exig_0480 | GntR family |
| Transcriptional regulator, DeoR family | Exig_1056, Exig_1150, Exig_1462, Exig_2269, Exig_0829, Exig_2742 | DeoR family |
| Transcriptional regulator, Crp/Fnr family | Exig_0595 | Crp/Fnr family |
| Transcriptional regulator, BadM/Rrf2 family | Exig_1556, Exig_1110, Exig_1218, Exig_1209, Exig_2080, Exig_0175, Exig_1703 | BadM/Rrf2 family |
| Transcriptional regulator, AsnC family | Exig_1527, Exig_2299 | AsnC family |
| Transcriptional regulator, ArsR family | Exig_1423, Exig_2737, Exig_1473, Exig_0544 | ArsR family |
| Transcriptional regulator, AraC family | Exig_2502 | AraC family |
| Transcriptional regulator, AbrB family | Exig_1457, Exig_0031 | AbrB family |
| SOS-response transcriptional repressor | Exig_1094 | LexA |
| Putative transcriptional regulator, PucR family | Exig_0679, Exig_2868 | PucR family |
| Cell envelope-related transcriptional attenuator | Exig_2563, Exig_2640, Exig_0812, Exig_2471, Exig_0479, Exig_0561 |  |
| PAS modulated sigma54 specific transcriptional regulator, Fis family | Exig_0914 | Fis family |
| Iron (metal) dependent repressor, DtxR family | Exig_0894 | DtxR family |
| Heat-inducible transcription repressor | Exig_0779 | HrcA |
| GTP-sensing pleiotropic transcriptional repressor CodY | Exig_1883 |  |
| Cyclic nucleotide-binding protein | Exig_0160 |  |

Table S2. Fold-change (log odds score) of *Exiguobacterium* *sibiricum* 255-15 grown at the indicated temperatures compared to 28^o^C.

| **ID** | **Gene Product** | **Protein** | **28^o^C versus** | | | |
| --- | --- | --- | --- | --- | --- | --- |
|  |  |  | **10^o^C** | **-2.5^o^C** | | **40^o^C** |
| **General metabolism** | | | | | | |
| Exig1939 | Orotate phosphoribosyl transferase | PyrE | -3.9 | -5.6 | | -0.9 |
| Exig1940 | Orotidine 5’-phosphate decarboxylase | PyrF | -3.8 | -5.3 | | -0.8 |
| Exig1941 | Dihydroorotate dehydrogenase 1 | PyrD | -2.8 | -4.1 | | -1.0 |
| Exig1942 | dihydroorotate dehydrogenase electron transfer subunit | UbiB | -4.3 | -6.0 | | -0.7 |
| Exig1943 | Carbamoyl-phosphate synthase, large subunit, glutamine-dependent | CarB | -4.6 | -5.8 | | -0.9 |
| Exig1944 | Carbamoyl-phosphate synthase, small subunit | CarA | -4.1 | -7.0 | | -1.2 |
| Exig1945 | Dihydroorotase multifunctional complex type | PyrC | -3.8 | -5.1 | | -1.5 |
| Exig1946 | Aspartate carbamoyltransferase | PyrB | -3.9 | -5.4 | | -1.3 |
| Exig1947 | Xanthine/uracil permease | UraA | -2.6 | -4.7 | | -0.5 |
| Exig1948 | pyrimidine operon attenuation protein | PyrR | -2.2 | -3.9 | | -0.3 |
| Exig0457 | AICARFT/IMPCHase bienzyme | PurD | -1.1 | -0.4 | | 0.0 |
| Exig0456 | Phosphoribosylglycinamide formyltransferase | PurH | -1.2 | -1.6 | | 0.1 |
| Exig0455 | Phosphoribosylformylglycinamidine cyclo-ligase | PurN | -1.2 | -2.5 | | 0.4 |
| Exig0454 | Amidophosphoribosyl transferase | PurM | -1.0 | -2.8 | | 0.1 |
| Exig0453 | Phosphoribosylformylglycinamidine synthase II | PurF | -0.7 | -2.8 | | -0.2 |
| Exig0452 | Phosphoribosylformylglycinamidine synthase I | PurL | -1.3 | -2.3 | | -0.5 |
| Exig0451 | Phosphoribosylformylglycinamidine synthetase PurS | PurL | -0.8 | -1.6 | | -0.3 |
| Exig0450 | SAICAR synthetase | PurS | -0.8 | -3.4 | | -0.4 |
| Exig0449 | Adenylosuccinate lyase | PurC | -1.0 | -2.5 | | -0.8 |
| Exig0448 | Phosphoribosylaminoimidazole carboxylase, ATPase subunit | PurB | -0.6 | -2.6 | | -0.4 |
| Exig0447 | 1-(5-Phosphoribosyl)-5-amino-4-imidazole-carboxylate (AIR) carboxylase | PurK | -1.0 | -2.4 | | -1.1 |
| Exig3029 | Adenylosuccinate synthetase | PurA | -0.9 | -0.8 | | 0.3 |
| **Energy metabolism** | |  |  |  |  | |
| Exig2320 | NADH dehydrogenase | Ndh | -0.1 | -2.0 | -0.5 | |
| Exig2312 | NADH dehydrogenase | Ndh | -0.6 | -1.3 | 0.5 | |
| Exig1789 | Ubiquinone/menaquinone biosynthesis methyltransferase | UbiE | -0.8 | -2.2 | -0.9 | |
| Exig2816 | Nicotinate phosphoribosyltransferase related | PncB | 0.2 | 1.0 | 0.4 | |
| Exig0975 | oxidoreductase, putative |  | 0.3 | 3.2 | 0.9 | |
| Exig0937 | 2-polyprenyl-3-methyl-5-hydroxy-6-metoxy-1 4-benzoquinol methylase | UbiQ | -0.1 | -1.6 | -0.1 | |
| Exig2166 | electron transfer flavoprotein beta-subunit | FixA | -0.2 | 1.8 | -0.3 | |
| Exig2165 | electron transfer flavoprotein, alpha subunit | FixB | 0.3 | 2.4 | 0.3 | |
| Exig0686 | NADH-dependent flavin oxidoreductase | NemA | 0.2 | 1.2 | -0.1 | |
| Exig2600 | Rieske 2Fe-2S iron-sulfur protein |  | -0.2 | 3.6 | 0.0 | |
| Exig2677 | H+-transporting two-sector ATPase, gamma subunit | AtpD | 0.1 | -0.9 | -0.9 | |
| Exig2678 | ATP synthase F1, alpha subunit | AtpG | 0.1 | -1.6 | -1.8 | |
| Exig2679 | H+-transporting two-sector ATPase, delta (OSCP) subunit | AtpA | -0.5 | -1.2 | -1.7 | |
| Exig2680 | ATP synthase F0, subunit B | AtpH | -0.2 | -1.1 | -1.0 | |
| Exig2681 | ATP synthase F0, C subunit | AtpF | 0.2 | -1.3 | -1.2 | |
| Exig2713 | 6,7-dimethyl-8-ribityllumazine synthase | RibH | -0.9 | 2.0 | 0.4 | |
| Exig2714 | 3,4-Dihydroxy-2-butanone 4-phosphate synthase:GTP cyclohydrolase II | RibB | 0.3 | 2.5 | 0.5 | |
| Exig2715 | Lumazine-binding protein | RibC | 0.3 | 2.2 | 0.3 | |
| Exig2716 | Riboflavin biosynthesis protein RibD | RibD | -0.9 | 2.3 | 1.1 | |
| Exig1986 | cytochrome caa3 oxidase subunit III | CyoC | -1.7 | -2.2 | -1.2 | |
| Exig1987 | cytochrome c oxidase subunit I | CyoB | -1.2 | -2.0 | -1.2 | |
| Exig1988 | cytochrome c oxidase subunit II | CyoA | -0.5 | -4.1 | -1.7 | |
| Exig1989 | Protoheme IX farnesyltransferase | CyoE | -1.3 | -2.0 | -0.5 | |
| Exig0060 | Dihydropteroate synthase | FolP | -0.3 | 1.1 | -0.6 | |
| Exig0061 | Dihydroneopterin aldolase family:Dihydroneopterin aldolase | FolB | 0.5 | 2.2 | 0.5 | |
| Exig1742 | Dihydrofolate reductase | FolA | 0.2 | 3.0 | -0.6 | |
| **Carbohydrate metabolism** | |  |  |  |  | |
| Exig0205 | glycerol-3-phosphate dehydrogenase | GlpA | 0.5 | 2.6 | 1.1 | |
| Exig1091 | Glycerol kinase | GlpK | 0.6 | 2.4 | -0.3 | |
| Exig1092 | Aquaporin | GlpF | 1.7 | 3.8 | 0.1 | |
| Exig0716 | glycerol-3-phosphate dehydrogenase, aerobic | GlpA | -0.2 | -0.6 | 2.0 | |
| Exig1820 | simple sugar transport system permease protein |  | -1.1 | -2.3 | -1.5 | |
| Exig1821 | sugar ABC transporter (permease) |  | 0.1 | -1.6 | -0.4 | |
| Exig2306 | Phosphoenolpyruvate-protein phosphotransferase | PtsA | 1.1 | -0.1 | -1.5 | |
| Exig2308 | PTS system, glucose-specific IIBC component | PtsG | -0.4 | -1.0 | 0.2 | |
| Exig0212 | glucose uptake protein | GlcU | -0.7 | 1.3 | 1.9 | |
| Exig0211 | glucose 1-dehydrogenase | gdh | 0.0 | 2.5 | 2.3 | |
| Exig2914 | fructokinase | RbsK | -0.3 | -0.9 | 0.3 | |
| Exig2912 | PTS system, sucrose-specific IIBC component | PtsG | -0.6 | -2.6 | -1.1 | |
| Exig2352 | butyryl-CoA dehydrogenase | CaiA | 0.4 | 2.7 | -0.4 | |
| Exig2285 | Phosphoenolpyruvate carboxykinase (ATP) | PckA | 0.2 | 1.9 | 0.1 | |
| Exig2267 | Proton/sugar symporter |  | -1.1 | -1.1 | -1.4 | |
| Exig0870 | L-lactate dehydrogenase | Mdh | 0.8 | 3.1 | 1.6 | |
| Exig0856 | Na+/phosphate symporter | NptA | 1.6 | 3.9 | 1.4 | |
| Exig0751 | maltosaccharide ABC transporter, permease | MalG | 0.0 | 2.9 | 1.2 | |
| Exig0750 | maltosaccharide ABC transporter, permease protein | UgpA | -0.3 | 1.6 | 1.5 | |
| Exig0748 | Sodium/hydrogen exchanger | KefB | 0.1 | 1.4 | 0.3 | |
| Exig0715 | Glycerol kinase | GlpK | -3.0 | -0.4 | -0.5 | |
| Exig0713 | Aquaporin/Glycerol uptake | GlpF | -1.0 | 1.3 | -0.9 | |
| Exig0265 | Alpha amylase, catalytic subdomain | AmyA | -0.1 | 0.3 | 1.7 | |
| Exig0370 | transcriptional repressor of the xylose operon |  | 0.0 | 2.3 | -0.3 | |
| Exig0378 | UDP-glucose 4-epimerase | GalK | -0.3 | 0.9 | 0.2 | |
| Exig0380 | aldose 1-epimerase | GalT | -0.6 | 1.1 | 0.2 | |
| Exig0414 | glycerol dehydrogenase | ARA1 | -0.6 | -2.0 | 0.5 | |
| Exig0506 | PTS system, N-acetylglucosamine-specific IIBC component |  | 1.0 | 1.4 | -0.6 | |
| Exig0505 | Phosphotransferase system IIC components glucose/maltose/N-acetylglucosamine-specific |  | 0.7 | 3.8 | 0.2 | |
| Exig2496 | Sugar-specific permease, EIIA 1 domain |  | 3.0 | -0.9 | 0.2 | |
| Exig2537 | Alpha amylase, catalytic subdomain | AmyA | 0.6 | -2.6 | 0.3 | |
| Exig1692 | Alpha amylase, catalytic subdomain | AmyA | 1.5 | 2.6 | 1.0 | |
| Exig1739 | Alpha amylase, catalytic subdomain | AmyA | 0.0 | 3.0 | -0.3 | |
| Exig0606 | Pyruvate-formate lyase | PflD | 0.9 | 1.9 | 1.7 | |
| Exig1030 | pyruvate ferredoxin oxidoreductase, alpha subunit | PorA | -1.2 | -2.4 | 0.1 | |
| **Amino acid metabolism** | |  |  |  |  | |
| Exig2819 | phosphoglycerate mutase | GmpB | -0.3 | 0.9 | 0.2 | |
| Exig2816 | Nicotinate phosphoribosyltransferase related | PncB | 0.9 | 1.0 | 0.4 | |
| Exig2809 | Alanine racemase region | Alr | -0.5 | -1.0 | 0.2 | |
| Exig2343 | cystathionine beta-lyase | MetC | -2.6 | 1.1 | -0.4 | |
| Exig2344 | cysteine synthase | CystK | -3.1 | 0.3 | -0.7 | |
| Exig0204 | probable amino-acid transporter transmembrane protein | | 0.5 | -1.6 | 0.8 | |
| Exig2918 | Amino acid/peptide transporter |  | -1.1 | -1.4 | -0.3 | |
| Exig2369 | Histidinol phosphate phosphatase | HisJ | 0.4 | 1.5 | -0.3 | |
| Exig2368 | ATP phosphoribosyltransferase involved in histidine biosynthesis | HisZ | -0.5 | 1.3 | 0.0 | |
| Exig2367 | ATP phosphoribosyltransferase | HisG | -0.7 | 1.4 | 0.5 | |
| Exig2366 | Histidinol dehydrogenase | HisD | -0.9 | 1.1 | 0.1 | |
| Exig2365 | histidinol-phosphate aminotransferase | HisC | -0.6 | 1.2 | 0.3 | |
| Exig2364 | imidazoleglycerol-phosphate dehydratase | HisB | -0.6 | 1.5 | | 0.3 |
| Exig2363 | Imidazole glycerol phosphate synthase, glutamine amidotransferase subunit | HisH | -0.5 | 0.9 | | 0.3 |
| Exig2362 | Phosphoribosylformimino-5-aminoimidazole carboxamide ribotide isomerase | HisA | -0.6 | 1.4 | | 0.2 |
| Exig2361 | Imidazoleglycerol-phosphate synthase | HisF | -0.6 | 1.8 | | 0.4 |
| Exig2360 | phosphoribosyl-ATP pyrophosphatase/phosphoribosyl-AMP cyclohydrolase | HisI | -0.6 | 1.6 | | 0.3 |
| Exig2359 | phosphoribosyl-ATP pyrophosphatase |  | -0.7 | 1.0 | | -0.2 |
| Exig2356 | prolyne dehydrogenase |  | 0.7 | 1.1 | | -0.8 |
| Exig1032 | L-threonine 3-dehydrogenase | Tdh | -1.1 | -1.8 | | 0.0 |
| Exig0940 | Amino acid ABC transporter, permease protein, 3-TM region, His/Glu/Gln/Arg/opine | HisM | 2.0 | 1.7 | | -0.4 |
| Exig0939 | ABC-type amino acid transport | HisJ | 1.0 | 2.6 | | 0.1 |
| Exig0248 | Choline-glycine betaine transporter | BetT | 1.3 | -1.2 | | -0.7 |
| Exig2193 | Threonyl-tRNA synthetase, class IIa | ThrS | 0.1 | -1.8 | | -0.8 |
| Exig2184 | Phenylalanyl-tRNA synthetase alpha subunit | PheS | -0.2 | -1.6 | | -0.8 |
| Exig2183 | Phenylalanyl-tRNA synthetase, beta subunit | PheT | -0.3 | -1.5 | | -0.2 |
| Exig0827 | Glycyl-tRNA synthetase, alpha subunit | GlyQ | 0.0 | -2.1 | | -0.6 |
| Exig0658 | Gutamate-1-semialdehyde aminotransfer | HemL | 0.5 | 2.8 | | -0.8 |
| Exig0569 | Arginine biosynthesis | ArgJ | -0.1 | 3.2 | | 1.7 |
| Exig0519 | D-alanyl-D-alanine carboxypeptidase | VanY | 0.1 | 5.2 | | 0.8 |
| Exig0504 | Ornithine aminotransferase | PtsG | 0.7 | 2.7 | | -0.7 |
| Exig0502 | arginine ornithine antiporter |  | 1.3 | 1.4 | | -0.7 |
| Exig0494 | Glycine betaine/L-proline transport ATP-binding subunit | OpuBC | 1.4 | 2.9 | | 0.0 |
| Exig0493 | Polysaccharide deacetylase family protein |  | 1.8 | 2.5 | | -0.6 |
| Exig0488 | Dihydrodipicolinate reductase | DapD | 0.3 | 1.0 | | -0.3 |
| Exig0487 | Dihydrodipicolinate synthase subfamily | DapB | 1.4 | 1.6 | | -0.1 |
| Exig0486 | Aspartate-semialdehyde dehydrogenase, USG-1 related | DapA | 1.9 | 1.7 | | 0.1 |
| Exig1689 | Phosphoserine aminotransferase | SerC | 1.7 | 2.2 | | -1.0 |
| Exig1690 | D-3-phosphoglycerate dehydrogenase | SerA | 0.9 | 2.4 | | -0.3 |
| Exig1723 | Glutamate synthase, NADH/NADPH, small subunit 1 | GltD | 0.6 | 4.9 | | 1.1 |
| Exig1724 | glutamate synthase (NADPH) large chain |  | 1.5 | 4.5 | | 1.0 |
| **DNA replication, transcription and translation** | |  |  |  | |  |
| Exig2074 | Alanyl-tRNA synthetase, class IIc | AlaS | -0.2 | -1.5 | | -0.4 |
| Exig2235 | Tyrosyl-tRNA synthetase, class Ib | TyrS | -0.9 | -2.5 | | -0.4 |
| Exig2932 | Helicase, C-terminal:DEAD/DEAH box helicase, N-terminal | SrmB | 1.5 | 0.9 | | -0.7 |
| Exig1836 | Ribosome-binding factor A | RbfA | 0.4 | 0.9 | | -0.6 |
| Exig1837 | Initiation factor 2:Small GTP-binding protein domain | InfB | 0.5 | 1.1 | | -0.8 |
| Exig1838 | ribosomal protein, L7Ae family (50S ribosomal protein) | Rpl8A | 0.9 | 1.5 | | -0.4 |
| Exig1839 | Predicted nucleic-acid-binding protein implicated in transcription termination |  | 0.6 | 1.0 | | -0.6 |
| Exig1840 | Transcription termination factor NusA | NusaA | 0.7 | 1.2 | | -0.6 |
| Exig1850 | Elongation factor Ts | Tsf | -0.9 | -1.9 | | -0.5 |
| Exig1888 | Bacterial DNA topoisomerase I | TopA | -0.1 | -1.7 | | -0.9 |
| Exig1003 | Inosine guanosine and xanthosine phosphorylase | DeoA | 1.6 | 2.7 | | 1.4 |
| Exig1004 | pyrimidine-nucleoside phosphorylase | Pnp | 0.7 | 2.3 | | 1.0 |
| Exig0961 | Bacterial DNA topoisomerase III | TopA | 0.6 | 1.6 | | 0.2 |
| Exig3033 | DnaB helicase | DnaB | 0.0 | 2.3 | | 1.4 |
| Exig1951 | Isoleucyl-tRNA synthetase, class Ia | IleS | 1.0 | -1.9 | | -1.0 |
| Exig1971 | Ribosomal protein L32 | RpmF | 0.2 | -1.7 | | -0.1 |
| Exig2175 | Ribonuclease HIII | RnhC | -0.3 | 2.1 | 0.2 | |
| Exig2134 | Glutamate-1-semialdehyde-2,1-aminomutase | HemL | 0.5 | 1.7 | 0.6 | |
| Exig2133 | Valyl-tRNA synthetase, class Ia | ValS | 0.6 | -1.3 | -0.4 | |
| Exig2138 | Glutamyl-tRNA reductase | HemA | 0.1 | -1.0 | -0.3 | |
| Exig1162 | similar to Oligoendopeptidase F |  | -1.8 | 2.2 | 0.1 | |
| Exig1203 | DNA topoisomerase VI subunit A |  | -0.8 | 3.7 | 1.4 | |
| Exig0903 | NusB antitermination factor | NusB | -0.6 | -1.9 | -0.4 | |
| Exig0839 | Helicase, C-terminal:DEAD/DEAH box helicase, N-terminal | SrmB | 0.9 | 1.6 | -0.6 | |
| Exig2498 | Oligoendopeptidase F |  | 1.9 | 4.4 | 0.0 | |
| Exig2725 | Transcription termination factor Rho | Rho | 0.1 | 1.4 | -0.1 | |
| Exig1585 | Ribosomal protein L25 | RplY | 0.4 | 6.2 | 2.5 | |
| Exig1586 | Helicase, C-terminal:DEAD/DEAH box helicase, N-terminal | HepA | 1.1 | 0.7 | 2.0 | |
| Exig1589 | Gram positive topoisomerase IV, subunit B | GyrB | -0.1 | 4.4 | 2.6 | |
| Exig1590 | Gram positive topoisomerase IV, subunit A | GyrA | 0.0 | 1.1 | 0.5 | |
| Exig1817 | ATP-dependent DNA helicase RecQ | RecQ | -1.0 | -1.9 | 0.2 | |
| Exig0004 | RecF protein | RecF | 0.1 | 1.4 | -0.8 | |
| Exig1026 | RecA bacterial DNA recombination protein | RecA | 0.3 | 1.3 | 0.4 | |
| Exig2847 | DNA polymerase III epsilon subunit and related 3'-5' exonuclease | DnaQ | -0.5 | -1.2 | -0.6 | |
| Exig1912 | RecG-like helicase | RecG | 0.4 | 1.5 | -0.3 | |
| Exig0905 | Exonuclease VII, small subunit | XseB | -0.5 | -1.9 | -0.1 | |
| Exig0906 | Exonuclease VII, large subunit | XseA | -0.5 | -1.4 | -0.1 | |
| Exig0199 | Bacterial regulatory protein, MarR | MarR | 8.9 | 2.2 | 0.7 | |
| Exig1816 | Bacterial regulatory protein, MarR | MerR | -0.4 | -0.6 | 0.8 | |
| Exig1824 | Bacterial regulatory protein GntR, HTH | GntR | -0.4 | -1.8 | -1.1 | |
| Exig2400 | RNA polymerase sigma-54 factor | RpoN | 0.4 | 2.2 | 1.4 | |
| Exig1853 | Sigma-70 factor |  | 0.0 | -2.2 | 0.1 | |
| Exig1353 | Bacterial regulatory protein, MarR | MarR | 0.0 | 1.5 | -0.2 | |
| Exig1355 | Transcriptional regulator | MarR | -0.1 | -2.8 | -0.8 | |
| Exig1391 | Transcriptional regulator | GntR | 0.4 | -3.1 | 1.3 | |
| Exig1765 | Helicase, C-terminal:DEAD/DEAH box helicase, N-terminal |  | 0.3 | 1.5 | 0.5 | |
| **Miscellaneous observations** | | | | | | |
| Exig1854 | methylation of methyl-accepting chemotaxis protein KO: K03411 chemotaxis protein CheD | CheD | 0.0 | -2.1 | 0.3 | |
| Exig1855 | chemotactic methyltransferase inhibitor KO: K03410 chemotaxis protein CheC | CheC | -1.0 | -3.1 | -0.1 | |
| Exig1856 | CheW-like protein | CheW | -0.5 | -3.2 | 0.1 | |
| Exig1857 | CheW-like protein:ATP-binding region, ATPase-like:Hpt | CheA | -0.5 | -2.9 | 0.2 | |
| Exig1858 | putative flagellar biosynthesis protein FlhG KO: K04562 flagellar biosynthesis protein FlhG | FlhG | -0.3 | -3.0 | 0.3 | |
| Exig1859 | ATPas | FlhF | -0.8 | -3.7 | 0.0 | |
| Exig1860 | Flagellar biosynthesis protein FlhA | FlhA | -0.8 | -3.2 | 0.3 | |
| Exig1861 | Flagellar biosynthetic protein FlhB | FlhB | -1.0 | -3.7 | 0.3 | |
| Exig1862 | Flagellar biosynthesis protein FliR | FliR | -0.8 | -3.5 | 0.1 | |
| Exig1863 | Flagellar biosynthesis protein FliQ:Type III secretion protein HrpO | FliQ | -0.1 | -3.8 | 0.7 | |
| Exig1864 | Flagellar transport protein FliP | FliP | -1.0 | -3.3 | 0.3 | |
| Exig1865 | Flagellar biogenesis protein | FliO | -0.8 | -4.4 | 0.1 | |
| Exig1866 | Response regulator receiver | CheY | -0.6 | -3.3 | 0.4 | |
| Exig1867 | Surface presentation of antigens (SPOA) protein | CheC | -0.4 | -3.8 | 0.4 | |
| Exig1868 | Flagellar motor switch protein FliM | FliM | -1.2 | -3.7 | 0.0 | |
| Exig1869 | flagellar FliL protein KO | FliL | -0.5 | -3.1 | 0.5 | |
| Exig1873 | Flagellar hook-length control protein | FliK | -0.6 | -2.9 | 0.5 | |
| Exig1876 | ATPase FliI/YscN | FliI | -0.8 | -2.9 | 0.4 | |
| Exig1877 | Flagellar biosynthesis/type III secretory pathway protein | FliH | -0.5 | -2.2 | 0.5 | |
| Exig1878 | Flagellar motor switch protein FliG | FliG | -0.3 | -3.0 | 1.1 | |
| Exig1879 | Flagellar FliF M-ring protein | FliF | -0.9 | -3.6 | 0.3 | |
| Exig1880 | Flagellar hook-basal body complex protein FliE | FliE | -0.4 | -1.4 | 0.0 | |
| Exig1882 | Flagellar basal-body rod protein FlgB |  | -0.1 | -3.1 | 1.0 | |
| Exig2439 | Flagellar protein FliS | FliS | -0.2 | 0.7 | -0.1 | |
| Exig2440 | flagellar hook-associated protein 2 | FliD | 0.4 | 1.4 | 0.1 | |
| Exig2441 | flagellar protein | FlaG | 0.4 | 1.4 | 0.5 | |
| Exig2667 | flagellar hook-basal body protein | FlaG | 0.6 | -2.2 | -1.1 | |
| Exig2668 | flagellar basal-body rod protein KO | FlaG | -0.1 | -2.0 | -1.1 | |
| Exig2886 | Response regulator receiver | OmpR | 0.5 | 1.2 | 0.4 | |
| Exig2129 | Type II secretory pathway component PulJ | PulJ | -0.2 | -1.4 | -0.8 | |
| Exig2128 | Tfp pilus assembly protein | PilW | -0.3 | -0.4 | -0.3 | |
| Exig2124 | Type II secretory pathway ATPase | PulE | -0.4 | -1.6 | -1.2 | |
| Exig2123 | Pilus retraction protein PilT | PilT | -0.5 | -2.0 | -1.5 | |
| Exig2122 | type IV pilus assembly protein PilC | PilC | -0.8 | -1.9 | -1.1 | |
| Exig2121 | Tfp pilus assembly protein | PilE | -0.1 | -2.5 | -1.3 | |
| Exig2120 | signal peptidase | PulO | -0.1 | -1.5 | -1.3 | |

Table S3. Fold-change (log odds score) of *Exiguobacterium* *sibiricum* 255-15 grown at the indicated temperatures compared to 40^o^C.

| **ID** | **Gene Product** | **Protein** | **40^o^C versus** | | | |
| --- | --- | --- | --- | --- | --- | --- |
|  | |  | **28^o^C** | **10 ^o^C** | | **-2.5^o^C** |
| **Phosphate starvation** | |  |  |  | |  |
| Exig2901 | low-affinity inorganic phosphate transporter | PiT | -0.5 | -1 | | -1.9 |
| Exig0863 | PhoU | PhoU | 2.3 | 2.9 | | 1.8 |
| Exig0862 | Phosphate transport system permease protein 1 | PstB | 2.0 | 2.4 | | 1.8 |
| Exig0861 | Phosphate transport system permease protein 2 | PstA | 2.6 | 2.8 | | 1.6 |
| Exig0860 | Phosphate ABC transporter, permease protein PstC | PstC | 1.5 | 1.8 | | 1.7 |
| Exig0859 | Phosphate binding protein | PstS | 2.9 | 3.1 | | 1.7 |
| **Heat shock proteins** | |  |  |  | |  |
| Exig0782 | Heat shock protein DnaJ N-terminal | DnaJ | -0.3 | 0.6 | | 0.9 |
| Exig0781 | Molecular chaperone DnaK (Hsp70) | DnaK | 0.4 | 2.0 | 0.5 | |
| Exig0780 | molecular chaperone GrpE | GrpE | 0.8 | 1.9 | 1.5 | |
| Exig0779 | Negative regulator of class I heat shock protein | HrcA | 1.4 | 2.0 | 1.1 | |
| Exig0472 | ATPases with chaperone activity ATP-binding subunit | ClpA | 1.3 | 1.9 | 0.9 | |
| Exig2768 | Chaperonin Cpn60/TCP-1 | GroL | 1.7 | 3.3 | 2.4 | |
| Exig2769 | Chaperonin GroES (Hsp10) | GroS | 2.0 | 3.8 | 3.5 | |
